# Can IR Images of the Water Surface Be Used to Quantify the Energy Spectrum and the Turbulent Kinetic Energy Dissipation Rate?

**DOI:** 10.3390/s23229131

**Published:** 2023-11-12

**Authors:** Shelby L. Metoyer, Darek J. Bogucki

**Affiliations:** Department of Physical and Environmental Sciences, Texas A&M University—Corpus Christi, 6300 Ocean Dr, Corpus Christi, TX 78412, USA; dbogucki@tamucc.edu

**Keywords:** air–sea interaction, boundary layers, spectral analysis, near surface, channel flow, remote sensing

## Abstract

Near-surface oceanic turbulence plays an important role in the exchange of mass, momentum, and energy between the atmosphere and the ocean. The climate modifying the air–sea CO${}_{2}$ transfer rate varies linearly with the surface turbulent kinetic energy dissipation rate to the 1/4 power in a range of systems with different types of forcing, such as coastal oceans, river estuaries, large tidal freshwater rivers, and oceans. In the first part of this paper, we present a numerical study of the near-surface turbulent kinetic energy spectra deduced from a direct numerical simulation (DNS) compared to turbulent kinetic energy spectra deduced from idealized infrared (IR) images. The DNS temperature fields served as a surrogate for IR images from which we have calculated the underlying kinetic energy spectra. Despite the near-surface flow region being highly anisotropic, we demonstrated that modeled isotropic and homogeneous turbulence spectra can serve as an approximation to observed near-surface spectra within the inertial and dissipation ranges. The second part of this paper validates our numerical observations in a laboratory experiment. In this experiment, we compared the turbulent kinetic energy spectra near the surface, as measured using a submerged shear sensor with the spectra derived from infrared images collected from above the surface. The energy dissipation measured by the shear sensor was found to be within 20% of the dissipation value derived from the IR images. Numerically and experimentally, we have demonstrated that IR-based and remote measurement techniques of the aquatic near surface offer a potentially accurate and non-invasive way to measure near-surface turbulence, which is needed by the community to improve models of oceanic air–sea heat, momentum, and gas fluxes.

## 1. Introduction

**Figure 1:**
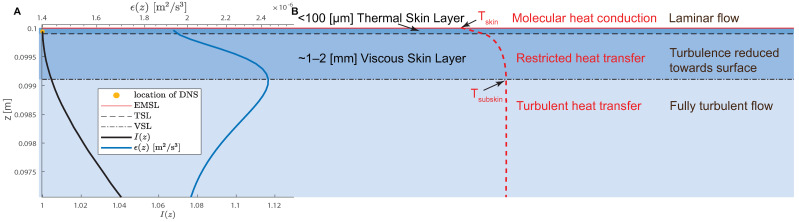
(**A**) The DNS-calculated vertical profiles of the energy dissipation rate, ϵ, and the large-scale anisotropy index, I(z), for run G09 of Pinelli et al. [4] (Dr. H. Herlina private communication). The vertical interval shown spans a depth of 3 mm from the flow surface. The TSL and the VSL extents are indicated by the dashed line and the dot-dashed line, respectively. The water-surface location is denoted by the blue line. We used the temperature and the velocity field from the second DNS grid layer, located at a depth of z=60 μm, indicated by the orange dot. The DNS temperature fields served as a surrogate for the IR images. For comparison, the IR penetration depth for IR wavelengths of 3.7–12 (μm) is ∼10–20 (μm) [5] and is shown as the EMSL. (**B**) A diagram representing near oceanic near-surface layers following Robinson [6]. Given the suppression of vertical turbulent transport within the TSL, the transport of heat there is predominantly through conduction, which requires a temperature gradient in the skin layer from the warmer to the colder fluid. The vertical temperature profile of the surface ocean is indicated by the red line. The temperature decreases from T${}_{\mathrm{skin}}$ at the surface to T${}_{\mathrm{subskin}}$ just below the VSL.

In the ocean, at any instance, there are multiple coexisting layers on the aqueous side of the air–sea boundary [1]. Prominent among them are the viscous skin layer (VSL), electromagnetic radiation skin layer (EMSL), and thermal skin layer (TSL) (Figure 1). The exchanges of heat, momentum, and gases, including greenhouse gases, are mediated by these layers and modulated by the near-surface turbulent kinetic energy dissipation (TKED) rates [2,3]. Accurate measurements of the turbulence at the ocean’s surface are, therefore, essential in understanding and quantifying the processes that control air–sea fluxes and their impact on climate dynamics.

The thickness of the TSL is typically around 0.1 mm [7]. A strong vertical temperature gradient is established within the TSL because of relatively poor molecular conduction efficiency compared to the turbulent counterpart [8]. Typically, the near-surface temperature difference, T${}_{\mathrm{skin}}-$T${}_{\mathrm{subskin}}$, ranges from −0.7 K at low wind speeds to −0.2 K at high wind speeds [5]. The VSL has a thickness of around 0.6–2 mm [9] and within it, viscosity becomes the dominant process [1].

The presence of such a strong near-surface vertical temperature gradient, in the ocean or other bodies of water, results in a surface populated by thermal structures that are readily visible in infrared (IR) images [10]. Thermal IR imaging is a natural choice for characterizing the air–sea interface, as the penetration depth for IR radiation at wavelengths of 3.7–12 μm is ∼10–20 μm [5]. We refer to the depth to which the IR radiation can travel as the EMSL (Figure 1). The IR observations of these structures have been used to detect wave breaking, quantify microscale wave breaking [11], or infer the gas flux [12] across an air–sea boundary. For a review of IR imaging methods used to obtain near-surface turbulence properties, see Chickadel et al. [13].

There was a notable attempt by Chickadel et al. [13] to quantify ϵ through TKE spectra. They estimated ϵ using an expression similar to Equation (20), except the constants multiplying $\epsilon^{2/3}K_{1}^{-5/3}$ were set to 0.5, where the expression was fitted to the TKE spectra and ϵ was estimated. They showed that the IR PIV-like technique, which we refer to as FIV, correlated well with in situ measurements. The mean velocities measured correlated with in situ mean velocities at $r^{2}>0.9$ and were able to get within 8–7% of the in situ estimated ϵ taken from 0.02 m beneath the surface. Furthermore, some of the most notable studies of this IR-based method [13,14,15] used very large-scale observation areas (with image widths ranging from 4 m to 250 m) with a relatively poor spatial resolution that did not adequately capture the small-scale velocity fluctuations that occurred on the order of millimeters, which are needed to resolve the inertial range of the TKE spectrum.

Unfortunately, there are few data documenting the near-surface aquatic spectra. Our measurements address the knowledge gap in observations of near-surface turbulence. Here, aided by the data set from the direct numerical simulations (DNS) of Pinelli et al. [4], we tested the hypothesis of whether the water-surface IR images can be used to quantify the near-surface TKED rates. The DNS simulation [4] was of an open channel flow with a free surface, and the temperature was represented by a scalar with a Schmidt number of 7. Our process of hypothesis testing consisted of three steps: (1) conversion of the near-surface temperature fields generated by the DNS runs to the one-dimensional (1D) velocity spectra with the aid of a PIV-like analysis known as Feature Image Velocimetry (FIV); (2) conversion of the DNS-generated 3D velocity fields to 1D velocity spectra; and (3) comparison of the space-time-averaged 1D velocity spectra.

In the final part of this paper, based on data collected during a laboratory experiment by Bogucki et al. [16], we compare the turbulent kinetic energy spectra near the surface, as measured using a submerged shear sensor with the spectra derived from infrared images collected from above the surface.

This paper is organized as follows. Our data analysis is carried out in terms of a homogeneous isotropic turbulent flow. Therefore, we briefly review the flow-relevant properties in Section 2. Section 3 introduces our data sets and relevant calculations. Section 4 discusses the data processing steps. In Section 5, we discuss the results, focusing on a comparison between the FIV and DNS spectra. Then, in Section 6, we present the laboratory measurements of the FIV spectra using an above-water IR camera and compare them to in situ measured spectra. We finish with a discussion in Section 7 and the conclusions in Section 8.

## 2. The Energy Spectra in the Homogeneous Isotropic Turbulence

Following Burchard and Umlauf [17] and Pope [18], we have summarized the properties of homogeneous isotropic turbulent flow essential to interpret our results. The flow velocity $u({\vec{r}}_{n})$ at the location ${\vec{r}}_{n}$ is denoted as $u\left({\vec{r}}_{n}\right)=u_{1}\left({\vec{r}}_{n}\right){\hat{x}}_{1}+u_{2}\left({\vec{r}}_{n}\right){\hat{x}}_{2}+u_{3}\left({\vec{r}}_{n}\right){\hat{x}}_{3}$. ${\hat{x}}_{i}$, with i=1…3, represents the unit vectors in the normed vector space. The ensemble-averaged two-point correlation, $R_{ij}\left(\vec{r}\right)$, at a spatial separation vector, $\vec{r}$, is given by:

(1) $$R_{ij}\left(\vec{r}\right)=\frac{1}{N}\sum_{n=1}^{N}u_{i}\left({\vec{r}}_{n}\right)u_{j}\left({\vec{r}}_{n}+\vec{r}\right)$$

where *N* is the total realization number. The energy spectrum tensor, $E_{ij}\left(\vec{K}\right)$, is obtained by taking the Fourier transform of $R_{ij}\left(\vec{r}\right)$ as:

(2) $$E_{ij}\left(\vec{K}\right)\equiv \frac{1}{{\left(2\pi \right)}^{3}}{\int \int \int }_{-\infty }^{\infty }R_{ij}\left(\vec{r}\right)e^{-i\vec{K}\vec{r}}d\vec{r}$$

where $\vec{K}=K_{i}{\hat{x}}_{i}+K_{2}{\hat{x}}_{2}+K_{3}{\hat{x}}_{3}$ is the vector in the wavenumber space. Given the length of the vector $\vec{K}$ as $K\;\equiv \;|\vec{K}|\;\equiv \;{\left(K_{1}^{2}+K_{2}^{2}+K_{3}^{2}\right)}^{1/2}$, we can then express the 3D energy spectrum as a scalar function, E(K), of a scalar argument, *K*, as (Pope [18]):

(3) $$E\left(K\right)={\;\int \int \int \;}_{-\infty }^{\infty }\frac{1}{2}E_{ii}\left(\vec{K}\right)\delta (|\vec{K}\left|-\kappa \right)d\vec{K}.$$

Typical turbulence measurements are carried out along a line segment, and thus the Fourier transform of the velocity/temperature measurements yields a 1D spectrum of the velocity/temperature. The 1D energy spectrum, $E_{ij}\left(K_{1}\right)$, is derived from Equation (2) by substituting the separation vector, ${\vec{r}}_{n}=x_{1}{\hat{x}}_{1}$, to yield:

(4) $$E_{ij}\left(K_{1}\right)\equiv \frac{1}{\pi }\int_{-\infty }^{\infty }R_{ij}\left(x_{1}{\hat{x}}_{1}\right)e^{-iK_{1}x_{1}{\vec{x}}_{1}}dx_{1}$$

where $K_{1}$ is the 1D wavenumber, which, here, is assumed to correspond to the longitudinal direction of ${\hat{x}}_{1}$. From Equation (4) with i=j=1, the 1D longitudinal spectrum, $E_{11}\left(K_{1}\right)$, is defined as:

(5) $$E_{11}\left(K_{1}\right)\equiv \frac{2}{\pi }\int_{0}^{\infty }R_{11}\left(x_{1}{\hat{x}}_{1}\right)\cos\left(K_{1}x_{1}\right)dx_{1}.$$

The 1D transverse energy spectrum, $E_{22}\left(K_{1}\right)$, such that the fluctuating velocities are parallel to the direction ${\hat{x}}_{2}$, can be expressed as:

(6) $$E_{22}\left(K_{1}\right)\equiv \frac{2}{\pi }\int_{0}^{\infty }R_{22}\left(x_{1}{\hat{x}}_{1}\right)\cos\left(K_{1}x_{1}\right)dx_{1}$$

The inverse transformation of $E_{11}$ (Equation (5)) is:

(7) $$R_{11}\left(x_{1}{\hat{x}}_{1}\right)=\int_{0}^{\infty }E_{11}\left(K_{1}\right)\cos\left(K_{1}x_{1}\right)dK_{1}$$

with an equivalent result for $E_{22}$ (Equation (6)). The kinetic energy components can be obtained from Equation (7), with the separation vector, $x_{1}=0$, as:

(8) $$\begin{array}{cc} \; & R_{11}\left(\vec{0}\right)=\langle u_{1}^{2}\rangle =\int_{0}^{\infty }E_{11}\left(K_{1}\right)dK_{1}\\ \; & R_{22}\left(\vec{0}\right)=\langle u_{2}^{2}\rangle =\int_{0}^{\infty }E_{22}\left(K_{1}\right)dK_{1}. \end{array}$$

The normalized autocorrelations of the longitudinal and transverse fluctuating velocities, *f* and *g*, are defined as:

(9) $$\begin{array}{cc} \; & f\left(x_{1}\right)=\frac{R_{11}\left(x_{1}{\hat{x}}_{1}\right)}{\langle u_{1}^{2}\rangle },\\ \; & g\left(x_{1}\right)=\frac{R_{22}\left(x_{1}{\hat{x}}_{1}\right)}{\langle u_{2}^{2}\rangle }, \end{array}$$

The longitudinal and transverse Taylor microscales, $\lambda_{f}$ and $\lambda_{g}$, can be derived [18] from Equation (9) as:

(10) $$\begin{array}{cc} \; & \lambda_{f}={\left[-\frac{1}{2}f^{\prime \prime }\left(0\right)\right]}^{-1/2}\\ \; & \lambda_{g}={\left[-\frac{1}{2}g^{\prime \prime }\left(0\right)\right]}^{-1/2} \end{array}$$

where for an isotropic turbulence, $\lambda_{g}=\lambda_{f}/\sqrt{2}$. $\lambda_{g}$ is related to ϵ as $\epsilon =15\nu u_{\mathrm{rms}}^{\prime 2}/\lambda_{g}^{2}$, where $u_{\mathrm{rms}}^{\prime }$ is the root mean square fluctuating velocity. The Taylor-scale Reynolds number, Reλ, can be expressed as:

(11) Reλ≡urms′λgν

$E_{11}\left(K_{1}\right)$ and $E_{22}\left(K_{1}\right)$ can thus be expressed as:

(12) $$\begin{array}{cc} \; & E_{11}\left(K_{1}\right)=\frac{2}{\pi }\langle {\tilde{u}}_{1}^{2}\rangle \int_{0}^{\infty }f\left(x_{1}\right)\cos\left(K_{1}x_{1}\right)dx_{1}\\ \; & E_{22}\left(K_{1}\right)=\frac{2}{\pi }\langle {\tilde{u}}_{2}^{2}\rangle \int_{0}^{\infty }g\left(x_{1}\right)\cos\left(K_{1}x_{1}\right)dx_{1} \end{array}$$

The relationship between the 1D and 3D energy spectra can be inferred from Equations (2) and (5) as:

(13) $$E\left(K\right)=\frac{1}{2}K^{3}\frac{d}{dK}\left(\frac{1}{K}\frac{dE_{11}\left(K\right)}{dK}\right)$$

and the inverse relationship as:

(14) $$E_{11}\left(K_{1}\right)=\int_{K_{1}}^{\infty }\frac{E(K)}{K}\left(1-\frac{K_{1}^{2}}{K^{2}}\right)dK$$

for the 1D transverse spectrum, $E_{22}\left(K_{1}\right)$, we have:

(15) $$E\left(K\right)=-K\left(\frac{dE_{22}\left(K\right)}{dK}+\int_{K}^{\infty }\frac{1}{K_{1}}\frac{dE_{22}\left(K_{1}\right)}{dK_{1}}dK_{1}\right)$$

with an inverse relationship as:

(16) $$E_{22}\left(K_{1}\right)=\frac{1}{2}\int_{K_{1}}^{\infty }\frac{E(K)}{K}\left(1+\frac{K_{1}^{2}}{K^{2}}\right)dK$$

and $E_{22}$ is related to $E_{11}$ by:

(17) $$E_{22}\left(K_{1}\right)=\frac{1}{2}\left(E_{11}\left(K_{1}\right)-K_{1}\frac{dE_{11}\left(K_{1}\right)}{dK_{1}}\right)$$

Furthermore, the 1D energy spectra, $E_{11}$ and $E_{11}$, are both directly related to the 3D spectra, E(K), by:

(18) $$E\left(K\right)=-K\frac{d}{dK}\left(\frac{1}{2}E_{11}\left(K\right)+E_{22}\left(K\right)\right).$$

**Figure 2:**
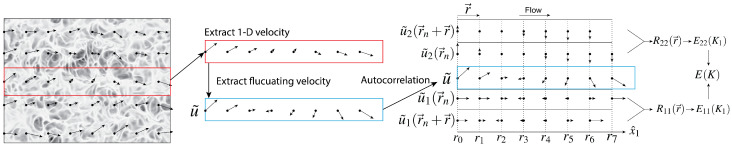
Illustration of the calculation of the 1D energy spectra from the velocity fields. We start with the 2D velocity field (overlaid here on the IR image) shown on the far-left side. From the 2D velocity field, we extract velocity vectors along the *x*-direction. Then, we extract the fluctuating velocity and use the appropriate autocorrelations to obtain the two-point correlations, $R_{11}$ and $R_{11}$. From the autocorrelations, we calculate the 1D energy spectra and estimate the 3D spectra. For details, see Section 4.1 ‘Calculations of 2D Flow Velocity from the DNS 2D Temperature Field’.

The process of calculating the velocity autocorrelation from the DNS data is illustrated in Figure 2.

The energy dissipation rate, ϵ, (or TKED) can be calculated from the 3D spectra, E(K), or 1D spectra, $E_{11}\left(K_{1}\right)$ and $E_{22}\left(K_{1}\right)$, or the equivalent dissipation spectra $D_{11}\left(K_{1}\right)$, $D_{22}\left(K_{1}\right)$, and D(K) as:

(19) $$\begin{array}{cc} \; & \epsilon =2\nu \int_{0}^{\infty }K^{2}E\left(K\right)dK\equiv \int_{0}^{\infty }D\left(K\right)dK\\ \; & \epsilon =15\nu \int_{0}^{\infty }K_{1}^{2}E_{11}\left(K_{1}\right)dK_{1}\equiv \int_{0}^{\infty }D_{11}\left(K1\right)dK_{1}\\ \; & \epsilon =\frac{15}{2}\nu \int_{0}^{\infty }K_{1}^{2}E_{22}\left(K_{1}\right)dK_{1}\equiv \int_{0}^{\infty }D_{22}\left(K_{1}\right)dK_{1} \end{array}$$

The Kolmogorov microscales are given as follows: $\eta \equiv {\left(\nu^{3}/\epsilon \right)}^{1/4}$ is the length microscale, $\tau \equiv {\left(\nu /\epsilon \right)}^{1/2}$ is the time microscale, and $\upsilon \equiv {\left(\nu \epsilon \right)}^{1/4}$ is the velocity microscale.

### Inertial and Far Dissipation Range Models

In the inertial range [18], the 3D and 1D velocity spectra follow:

(20) $$\begin{array}{cc} \; & E_{11}\left(K_{1}\right)=\frac{18}{55}K_{0}\epsilon^{2/3}K_{1}^{-5/3}\\ \; & E_{22}\left(K_{1}\right)=\frac{24}{55}K_{0}\epsilon^{2/3}K_{1}^{-5/3}\\ \; & E\left(K\right)=K_{0}\epsilon^{2/3}K^{-5/3} \end{array}$$

where $K_{0}$ is the Kolmogorov constant, 1.83 [19].

In the far dissipation wavenumber range, i.e., when Kη>7, researchers have observed that the energy spectrum depends on the flow Re number [20]. For flows with $Re_{\lambda }<70$ [20], the E(K) in the far dissipation wavenumber range has been observed as being characterized by a simple exponential wavenumber dependence, as predicted by Kraichnan [21]. Given the consistent observation of flows within the VSL, we assume that our analyzed DNS energy spectra, asymptotically in the far dissipation range, could be characterized by a simple exponential wavenumber dependence expressed as:

(21) $$E\left(K\right)\overset{\eta K\to \infty }{=}B\cdot e^{-a\eta K}$$

with B=8.4 and a=5.1 [22]. Using Equations (14) and (16), the respective 1D equivalent models of the exponential form (Equation (21)) for a flow characterized by a low Re number are:

(22) $$\begin{array}{cc} \; & E_{11}\left(K_{1}\right)=\frac{1}{2}B\cdot e^{-\xi }\left[\xi -1\right]-\frac{1}{2}B\cdot E_{\mathrm{in}}\left(\xi \right)\left[\xi^{2}-2\right]\\ \; & E_{22}\left(K_{1}\right)=\frac{1}{4}B\cdot e^{-\xi }\left[1-\xi \right]+\frac{1}{4}B\cdot E_{\mathrm{in}}\left(\xi \right)\left[\xi^{2}+2\right] \end{array}$$

where the scaled wavenumber, ξ, is expressed as $\xi =a\cdot \eta \cdot K_{1}$, and $E_{in}$ is the exponential integral [23].

## 3. Description of DNS Simulation

The DNS data used here were obtained by Pinelli et al. [4]. We used data from their run G09, which had a grid resolution of 1152×384×1152 corresponding to a solution of the Navier–Stokes equations for a free-surface open-water channel flow. The DNS domain size was 24H×H×6H in the *x*-, *y*-, and *z*-directions, respectively. The flow depth in dimensional units was H=0.1 m. The co- and cross-stream directions were denoted as *x* and *y*, respectively. The DNS data set used consisted of two sets of fields: the velocity and temperature fields taken at a near-surface location at the second computational layer beneath the surface, corresponding to a 60 μm dimensional depth, which is a fraction of the Kolmogorov length scale η. The DNS temperature field was represented by a scalar field with a Schmidt number of 7.

The free surface was assumed to be flat, thereby neglecting any possible influence of waves. In the vertical direction, a free-slip boundary condition at the free surface, z/H=1, and a no-slip boundary condition at the bottom were imposed. The temperature field had a surface layer that was kept at a constant temperature, whereas a zero-flux boundary condition was imposed on the bottom layer. The periodic boundary conditions were used in the co-stream (*x*, longitudinal) and cross-stream (*y*, transverse) directions for both the temperature and flow fields. A summary of the selected parameters characterizing the analyzed DNS run is presented in Table 1 under the heading ‘DNS’.

After the initialization of the DNS runs, the temperature field was introduced, and the DNS simulation was spun up until the fields reached a quasi-steady state [4]. Large-scale motion (LSM) was observed as elongated streaks in the co-stream direction of the fluctuating velocity field, as depicted in Figure 2e of Pinelli et al. [4]. Del Alamo and Jiménez [24] recommend a minimum of 10 wash-out times to capture LSM statistics. The wash-out time is the time it takes for the flow to traverse the entire DNS domain. In the DNS [4] and the data used here, the captured data corresponded to a little over two wash-out times, likely contributing to the increased statistical uncertainty of the analyzed DNS data.

### The DNS-Observed Near-Surface Flow Anisotropy and Energy Dissipation

The near-surface flow is typically anisotropic, and Pinelli et al. [4] quantified the large-scale flow anisotropy as:

(23) $$I\left(z\right)=\bar{\left(\langle uu\rangle +\langle vv\rangle +\langle ww\rangle )/(\langle uu\rangle +\langle vv\rangle \right)}$$

where u,v, and *w* are the flow velocity components, and here, 〈…〉 denotes the average over space and time. As the surface was approached, the vertical velocity component, *w*, decreased until it became zero at the surface, resulting in I(z=H)=1, as shown in Figure 1.

Pinelli et al. [4] calculated the (x,y) plane space-time-averaged TKE dissipation rate as $\epsilon \left(z\right)=\nu \langle \frac{\partial u_{i}^{\prime }}{\partial x_{j}}\frac{\partial u_{i}^{\prime }}{\partial x_{j}}\rangle$. The vertical profile of ϵ(z) is presented in Figure 1, with ϵ(z) characterized by a bump at z=0.099 m, corresponding to a depth of 1 mm. The presence of the ϵ peak was consistent with the near-surface vortex’s interaction with the free surface, as observed in the experiments of Gharib and Weigand [25].

## 4. DNS Data Processing

### 4.1. Calculations of 2D Flow Velocity from the DNS 2D Temperature Field

DNS temperature fields from a 60 μm depth (second computational grid point) were used to represent the IR camera-retrieved near-surface water temperature. We used Feature Image Velocimetry (FIV), as presented in Metoyer et al. [26], which is similar to Particle Image Velocimetry (PIV), to convert the DNS temperature field to a 2D velocity field time series. The process began with the conversion of the non-dimensional temperature field, from the DNS to normalized 8-bit non-compressed images. The newly created time-series images were then sequentially windowed. The windowing process divided the 1152 × 1552 pixel image pairs into a grid of 73 × 73 windows. Each window was equally sized, covering an area of 60 pixels${}^{2}$, and equally spaced 30 pixels apart. The window pairs were then cross-correlated, and a vector field was retrieved.

The obtained vector field was subjected to a standard deviation filter, eliminating values beyond five standard deviations, along with the implementation of the universal outlier detector for PIV data [27]. This filtering process resulted in gaps within the data set that required interpolation for a more comprehensive analysis. The interpolation technique employed followed the approach described by Halim and Kumar [28] and accounted for approximately 2.6% of the derived velocities in the FIV data set. The near-surface temperature field from the DNS showed IR features (Figure 2) that were similar to those observed by an IR camera of an open channel flow. However, the fact that we were using the DNS temperature field as a proxy for the IR images resulted in an extreme increase in the resolution without any of the noise that is normally present in IR imaging of the near surface [26]. A summary of the selected flow parameters characterizing the analyzed FIV measurements *inferred from the DNS temperature field* are presented in Table 1 under the heading ‘FIV’.

### 4.2. Calculations of 3D and 1D Energy Spectra from 2D Flow Fields

The procedure for converting the 2D velocity fields, obtained either directly from the DNS flow or the temperature-processed data, to their homogeneous and isotropic 1D or 3D equivalent velocity spectra was as follows. We started with the velocity field shown on the far-left side of Figure 2. From the 2D velocity field, we extracted one row as a 1D velocity series. Then, we extracted the fluctuating velocity as $\tilde{u}$, which was then fed into the autocorrelation algorithm, Equation (1). The 1D fluctuating velocity, $\tilde{u}$, had the components ${\tilde{u}}_{1}\left({\vec{r}}_{n}\right)$ in the ${\hat{x}}_{1}$-direction and ${\tilde{u}}_{2}\left({\vec{r}}_{n}\right)$ in the ${\hat{x}}_{2}$-direction.

The 1D autocorrelation function, $R_{11}\left(\vec{r}\right)$, in the ${\hat{x}}_{1}$-direction was calculated as $R_{11}\left(\vec{0}\right)=\frac{1}{N}\sum_{n=0}^{N-1}{\tilde{u}}_{1}{\left({\vec{r}}_{n}\right)}^{2}$, $R_{11}\left(1{\hat{x}}_{1}\right)=\frac{1}{N}\sum_{n=0}^{N-1}{\tilde{u}}_{1}\left({\vec{r}}_{n}\right){\tilde{u}}_{1}\left({\vec{r}}_{n}+1\hat{x}\right)$, etc. From the 1D autocorrelations functions, $R_{11}$ and $R_{22}$, the 1D spectra, $E_{11}\left(K_{1}\right)$ and $E_{22}\left(K_{1}\right)$, were calculated using Equations (5) and (6), respectively. From $E_{11}\left(K_{1}\right)$ and $E_{22}\left(K_{1}\right)$, with the aid of Equation (18), we calculated the 3D energy spectra E(K). All sets of energy spectra were finally averaged over space and time.

## 5. Results—DNS-Based Spectra

To quantify the faithfulness of the derived energy spectra and energy dissipation from IR, we compared the 1D and 3D velocity spectra deduced from the IR images to the DNS velocity spectra. Our analysis focused on the inertial and far dissipation spectral ranges, as that part of the energy spectrum determines the fidelity of IR near-surface energy dissipation. In the plots and the text, ‘DNS’ refers to the spectra obtained from the DNS flow data, whereas ‘FIV’ refers to the spectra obtained from the DNS temperature fields after processing them using the FIV algorithm.

### 5.1. Energy Spectra

**Figure 3:**
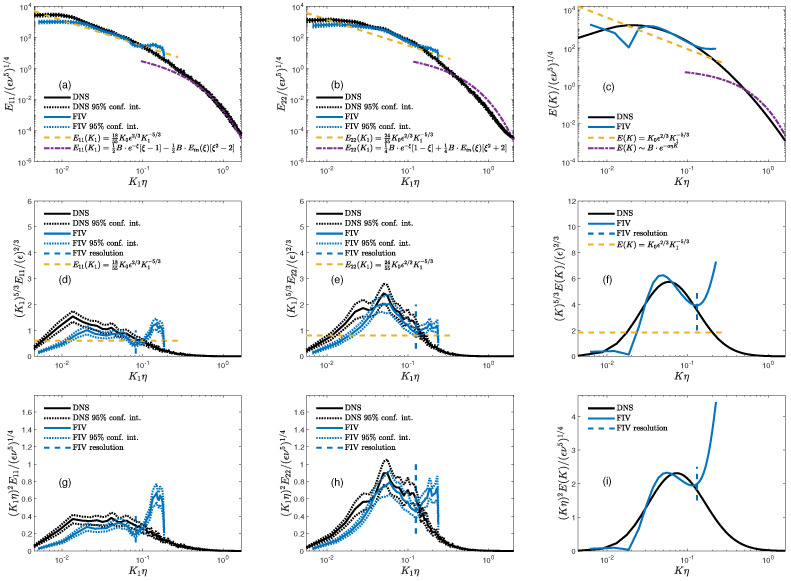
(**a**–**c**) The space-time-averaged DNS and FIV spectra presented as $E_{11}\left(K_{1}\right)$, $E_{22}\left(K_{1}\right)$, and E(K) (Equations (5), (6) and (18)), compared to their homogeneous and isotropic models (Equations (20) and (21)). The compensated spectra are such that the inertial range model (Equation (20)) is a horizontal line segment plotted in (**d**–**f**). Plots (**g**–**i**) show the dissipation spectra $D_{11}\left(K_{1}\right)$, $D_{22}\left(K_{1}\right)$, and D(K) (Equation (19)).

In Figure 3, the 1D TKE spectra, $E_{11}\left(k\right)$ and $E_{22}\left(k\right)$, as well as the 3D spectra, E(K), are presented. The subplots (a), (b), and (c) (Figure 3) present the DNS (black line) and FIV (blue line) spectra, accompanied by their respective 95% confidence intervals, compared to the model spectra of either the longitudinal, $E_{11}$ (*x*-direction); transverse, $E_{22}$ (*y*-direction), or 3D E(k) normalized model energy spectra. The model spectra represent the homogeneous and isotropic turbulent flows as in Equation (20) (yellow line) and Equation (22) (magenta line).

The spectra within the low wavenumber $K_{1}\eta <0.02$ have larger statistical uncertainty due to the temperature field being in a quasi-steady state and only having a little more than 2 wash-out times’ worth of captured data—recall that Del Alamo and Jiménez [24] suggest 10 wash-times to obtain reliable statistics of the LSM. Within the wavenumber interval $0.02<K_{1}\eta <0.1$ (Figure 3a–c), the FIV spectra roughly follow the DNS spectra, with the best fit exhibited by $E_{11}\left(K_{1}\right)$. The DNS data show also a fairly short range of the inertial range roughly covering the interval $0.01<K_{1}\eta <0.1$ (Figure 3a). The FIV spectra at large wavenumbers were limited by the FIV retrieval noise to the largest wavenumbers bound by $K_{1}\eta <0.1$.

Interestingly, the $E_{11}\left(k\right)$ spectra (1D spectra in the *x*-direction) were in fair agreement with the model spectra for either the inertial (for the ‘DNS’ and ‘FIV’ data sets) or the dissipation range (for the ‘DNS’ data). We posit that the large degree of agreement between the modeled and measured spectra in the *x*-direction was due to the length of the computational DNS domain in that direction. In general, the $E_{11}$ spectra presented in Figure 3a provide evidence that the near-surface turbulent flows can be approximated by homogeneous and isotropic model spectra, notably well in the far dissipation range. This can be attributed to the ability of the fluctuating part of the flow to retain a significant degree of isotropy and homogeneity while deeply within the VSL. We plan on testing this hypothesis in future work.

### 5.2. Inertial Part of the Spectrum

Figure 3d–f present the 1D and 3D compensated spectra, such that the modeled inertial range is a horizontal line segment. The FIV spectra followed the DNS spectra (Figure 3d,e), with $E_{11}\left(K_{1}\right)$ most closely following the DNS data over the interval $0.02<K_{1}\eta <0.1$. The DNS and FIV 1D spectra show a systematic departure from the inertial part of the spectrum, with $E_{11}\left(K_{1}\right)$ showing the smallest deviation from the isotropic and homogeneous turbulence model spectra (Equation (20)). This departure from the isotropic and homogeneous spectra was consistent with the observations of Bogucki et al. [16]. In general, within the VSL, the shift of the spectra from their isotropic and homogeneous turbulence model was expected, as the flow within the VSL was characterized by a low Reynolds number, with the majority of the TKE dissipation (3D spectra) taking place at wavenumbers around ηK≃0.1. The experiments by Bogucki et al. [16] additionally documented that within the VSL, the extent of the inertial range and the departure from the modeled isotropic and homogeneous spectra were proportional and inversely proportional to the distance to the surface, respectively.

### 5.3. Energy Dissipation Spectra

The 1D and 3D dissipation spectra are presented in Figure 3g–i. In general, the FIV data accurately captured the energy dissipation peak, as shown in Figure 3g–i. In the 3D dissipation DNS spectra (Figure 3i), the dissipation peak was located at Kη=0.07, which was at a lower wavenumber compared to the low Reynolds number simulations of Bogucki et al. [22], where the dissipation peak was found at Kη=0.2. The 1D DNS spectral shape closely followed their FIV analog (Figure 3g,h), with the FIV spectra exhibiting somewhat smaller values, resulting in the underestimation of the energy dissipation when comparing the FIV and DNS ϵ values.

### 5.4. Comparison of DNS-Observed and FIV-Deduced Small-Scale Flow Properties

We observed that the homogeneous and isotropic spectra, when applied to our DNS or FIV data from within the VSL layer, did a remarkably good job in describing the flows within the inertial or dissipation ranges, considering that the mean flow was quasi-two-dimensional, as illustrated in Figure 1. The FIV-based measurements of $Re_{\lambda }$ (Equation (11)) were smaller than their DNS counterparts by roughly a factor of 3/2. This was due to the difference in their Taylor microscales (Equation (10)) and $u_{\mathrm{rms}}^{\prime }$ (Table 1).

The FIV energy dissipation was smaller than the DNS dissipation by roughly a factor of 2/3, underscoring the fact that the FIV dissipation spectra were smaller than their DNS counterparts (Figure 3a,b). This discrepancy in the FIV and DNS dissipations resulted in a disparity between the turbulent microscale parameters (Table 1).

## 6. Laboratory Experiment to Measure IR-Based and In Situ Dissipation Spectra

In the preceding section of our paper, we demonstrated the successful retrieval of surface TKE dissipation using numerically derived IR images of the water surface, processed using the FIV approach. To validate this claim in a laboratory setting, we carried out an experiment in a turbulent flume at the University of Miami’s RSMAS facility, as detailed in [16]. In this experiment, we positioned an off-shelf IR camera approximately 0.5 m above the water surface to record a video stream of the water surface. We utilized this time series of water-surface IR images via the FIV approach to derive the transverse dissipation spectra, denoted as $D_{22}\left(K_{1}\right)$ (see Equation (19)).

To verify the near-surface TKE dissipation measured with IR/FIV, we utilized a commercial micro-shear profiler (VMP200) to obtain the TKE dissipation spectra in the water. The VMP200 was positioned at a depth of 0.9 cm below the water surface, as described by Bogucki et al. [16]. The VMP200 instrument inherently provides one-dimensional $D_{22}\left(K_{1}\right)$ spectra, as noted by Bogucki et al. [16]. Both measurements, VMP200 and IR, yielded $D_{22}\left(K_{1}\right)$ spectra at a fixed location, which were averaged over time. The IR measurements were taken at the same location as the VMP200 measurements, with the VMP200 data collected at approximately the center frame of the IR camera’s field of view.

**Figure 4:**
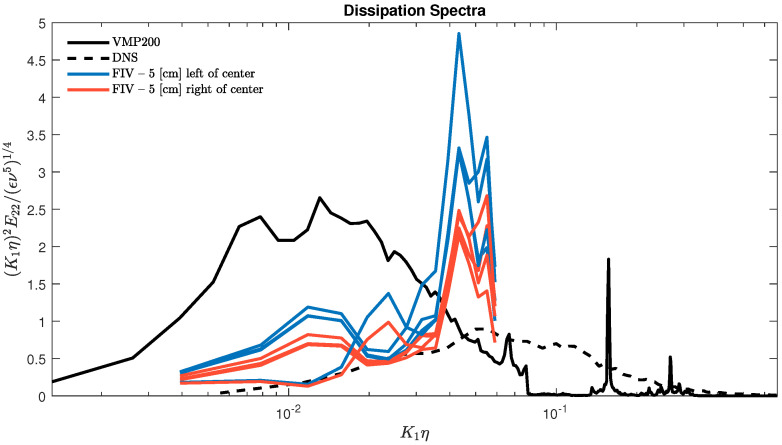
The transverse 1D dissipation spectra for the laboratory measurements of the VMP200 (black line) taken at 0.9 cm beneath the surface and FIV (blue and red lines) measurements taken from the surface, along with the DNS (black dashed line) transverse 1D dissipation spectra. The FIV measurements taken from the windows 5 cm to the left and right of the center of the image are shown in blue and red, respectively. The FIV processing parameters were then varied for each spectra shown.

The non-dimensional TKE dissipation spectra obtained through laboratory measurements using FIV/IR and VMP200 are illustrated in Figure 4. These measured spectra are presented alongside the transverse non-dimensional spectrum calculated from the DNS velocity field. In Figure 4, the black line represents the in-water VMP200-measured non-dimensional transverse spectrum, and the black dashed line corresponds to the non-dimensional transverse DNS spectrum (Equation (6)). To gain insight into the sensitivity of the IR/FIV-derived spectra to the FIV processing parameters and the pixel location within the IR camera image, a set of FIV dissipation spectra was plotted, as shown in Figure 4. The blue lines represent the IR FIV measurements taken from approximately 5 cm to the left of the center of the IR image, whereas the red lines represent measurements taken from approximately 5 cm to the right of the center of the IR image. The variability in the blue/red line spectra reflects the dissipation spectra variability over the IR camera footprint. For each red and blue line, we further varied the key parameters within the FIV algorithm to estimate their impact on the IR-derived dissipation spectra, as shown in Figure 4.

The VMP200- and FIV/IR-retrieved energy dissipation spectra were obtained from 0.9 cm and tens of micrometers below the surface, respectively. The VMP200-measured ϵ was $1.4\times 10^{-5}$ m${}^{2}$/m${}^{3}$, whereas the values of the FIV-measured ϵ calculated from the FIV spectra (refer to Figure 4) ranged from $0.6\times 10^{-5}$ to $1.1\times 10^{-5}$ m${}^{2}$/m${}^{3}$. The 95% confidence interval of the spectral FIV measurements was extremely small, given the 10 min of IR video and the fact that each spectrum was calculated from multiple points with over 1200 degrees of freedom. Therefore, the 95% confidence interval is not presented, as it does not account for the variance in the FIV spectra.

### Comparison of IR and In Situ-Measured Energy Dissipation Spectra in the Laboratory Experiment

The VMP200-measured dissipation peak, as shown in Figure 4, ranged from $K_{1}\eta =0.3\times 10^{-2}$ to $K_{1}\eta =9\times 10^{-2}$, with the dissipation peak situated at approximately $K_{1}\eta =1.5\times 10^{-2}$. The IR/FIV reported a dissipation peak ranging from roughly $K_{1}\eta =4\times 10^{-2}$ to $K_{1}\eta =7\times 10^{-2}$, with the dissipation peak located at around $K_{1}\eta =5\times 10^{-2}$. The IR/FIV’s measured dissipation peak, as shown in Figure 4, was somewhat larger than that of the VMP200. This observation is generally consistent with the findings of Bogucki et al. [16], who noted that dissipation peaks tend to become narrower and taller as we approach the surface.

We propose that this trend was related to dissipation occurring at very specific wavenumbers, which is linked to the near-surface flow becoming characterized by a shortening of its inertial range.

Bogucki et al. [16] also observed that the dissipation peak location remained invariant with depth, a fact not reflected in Figure 4. In general, the observations presented here support the notion that IR oceanic surface measurements can provide realistic TKE dissipation estimates. However, the disparity in the VMP200 and IR/FIV spectra, especially at large wavenumbers, needs to be addressed in a more comprehensive experiment. Our future work will focus on understanding the nature of large wavenumber noise and attempting to mitigate it.

## 7. Discussion

The community relies on similarity scaling to estimate surface boundary layer turbulence [29] in a variety of observational, analytical, and modeling pursuits, aiming to quantify oceanic air–sea fluxes of heat, momentum, and gas due to the inherent challenges of observation and representation of turbulence. The ability of the IR camera to remotely derive oceanic surface energy spectra and turbulent dissipation would facilitate improved global characterization of oceanic fluxes, for example, by re-analyzing existing IR surface images, such as those collected by the Saildrone fleet, [30].

### 7.1. FIV Measurements of DNS-Based Spectra

As illustrated in Figure 3, the FIV spectra exhibit a departure from the DNS spectra once they surpass wavenumbers corresponding to the FIV window size (indicated by the blue dashed line and labeled as ‘FIV resolution’). This divergence is notably more pronounced in the FIV longitudinal spectra (Equation (5)) compared to the FIV transverse spectrum (Equation (6)). It remains uncertain whether this discrepancy solely originates from the FIV method or if it stems from aspects within the DNS data set itself. It is important to emphasize that this was not a direct apples-to-apples comparison. The DNS spectra were computed based on the DNS velocity field, whereas the FIV measurements were conducted on the temperature field that experiences displacement due to the velocity field.

To gain a comprehensive understanding of the nature of this high-frequency noise, a detailed investigation of the core of FIV, particularly the direct cross-correlation, is essential. This inquiry aims to determine the point at which the high-frequency velocity fluctuations become unmeasurable for a given FIV window size.

It is worth noting that the DNS-based FIV spectra were calculated spatially (i.e., correlated over space), whereas the FIV spectra measured in the laboratory were temporal (i.e., correlated over time). Unfortunately, the acquisition time of the DNS was insufficient to adequately capture the temporal spectra and enable a meaningful comparison between the spatially correlated and temporally correlated spectra.

### 7.2. Laboratory FIV Measurements vs. In Situ and DNS Dissipation Spectra

The dissipation spectra obtained through IR measurements manifest a more pronounced and narrower dissipation peak in comparison to those acquired via the shear sensor. The laboratory data set presented in Figure 4 underscores the remarkable consistency in the retrieved FIV spectra when processed with diverse parameters, with the primary source of variation predominantly arising from the left–right divide.

At approximately $K_{1}\eta =5\times 10^{-2}$, the FIV dissipation spectra exhibit a steep ascent, aligning closely with the anticipated peak dissipation, as predicted by the DNS data. Remarkably, as depicted in Figure 4, we observe high-frequency noise resembling that of DNS-based FIV spectra. However, this noise primarily emerges after the energy dissipation peak, with nearly all right-of-center FIV spectra (red line—Figure 4), regardless of the chosen parameters, capturing the same dissipation peak. It is only after the peak that the FIV spectra begin to exhibit more pronounced deviations.

Regrettably, the dissipation peak in the FIV data occurs near the Nyquist frequency, resulting in an underestimation of ϵ. This stems from the truncation of part of the dissipation spectra at this particular frequency. Given the characteristics of the turbulent length and timescales, η and τ, there is a compelling need for an IR camera with higher resolution, expanded bit depth, and a faster frame rate to effectively measure oceanic flows.

## 8. Conclusions

Our observations demonstrated that, in principle, the FIV technique [26], when applied to DNS temperatures within the VSL, was remarkably successful in reproducing the underlying velocity spectra and dissipation spectra.

To validate the applicability of our approach, we conducted a laboratory experiment. The data collected in this experiment demonstrate that the FIV measurements were accurate in practical settings, with the TKE dissipation measurements being within 20% of the in situ measurements.

The work presented here underscores the intriguing potential of using ocean-surface IR images to study surface ocean turbulence properties, including energy spectra and turbulent kinetic energy dissipation. This measurement could address existing knowledge gaps and contribute to our understanding of global air–sea fluxes. Furthermore, the wealth of existing IR oceanic surface images could immediately benefit from this measurement technique.

However, more work must be undertaken to verify the FIV method under diverse conditions and gain a more comprehensive understanding of how accurately FIV measures the TKE and TKED spectra. A comprehensive study involving a combination of DNS and laboratory datasets and a more in-depth examination of the precise workings of FIV and its accuracy could prove fruitful in developing FIV into a more fully fledged measurement scheme with reduced errors in spectral measurements. This pursuit is particularly worthwhile given that we have now demonstrated the feasibility of obtaining the dissipation spectral range from the IR measurements, the spectral range from which ϵ is calculated.

## Figures and Tables

**Table 1:** Comparison of turbulent microscale parameters derived from the simulated temperature (FIV) of the velocity (DNS) fields. The calculations were carried out using several spectra—$E_{11}$, $E_{22}$, or E(K)—to obtain the Kolmogorov length scales η, time scale τ, velocity scale υ, Taylor microscale λ (Equation (10)), Taylor-scale Reynolds number $Re_{\lambda }$ (Equation (11)), and TKED rate ϵ (Equation (19)).

**DNS**						
	**η (m)**	**τ (s)**	**υ (m/s)**	**λ (m)**	Reλ	**ϵ (m^2^/s^3^)**
$E_{11}$	1.09 × $10^{-3}$	1.18	9.20 × $10^{-4}$	2.61 × 10${}^{-2}$	149	7.17 × $10^{-7}$
$E_{22}$	1.33 × $10^{-3}$	1.78	7.50 × $10^{-4}$	3.04 × 10${}^{-2}$	134	3.16 × $10^{-7}$
E(K)	1.26 × $10^{-3}$	1.59	7.94 × $10^{-4}$	3.62 × 10${}^{-2}$	213	3.98 × $10^{-7}$
**FIV**						
	**η (m)**	**τ (s)**	**υ (m/s)**	**λ (m)**	Reλ	**ϵ (m^2^/s^3^)**
$E_{11}$	1.15 × $10^{-3}$	1.31	8.72 × $10^{-4}$	2.03 × 10${}^{-2}$	81	5.79 × $10^{-7}$
$E_{22}$	1.49 × $10^{-3}$	2.21	6.72 × $10^{-4}$	2.79 × 10${}^{-2}$	90	2.04 × $10^{-7}$
*E* (K)	1.39 × $10^{-3}$	1.94	7.18 × $10^{-4}$	3.18 × 10${}^{-2}$	135	2.65 × $10^{-7}$

## Data Availability

The bulk of the data set was obtained from the direct numerical simulation (DNS) of an open channel flow with a shear-free surface generated at the Karlsruhe Institute of Technology, Karlsruhe, Germany, by Pinelli et al. [4]. The DNS includes the temperature, velocity, and divergence fields over 181 realizations. All fields are 1152 × 1152 and non-dimensional. The velocity fields can be dimensionalized by a velocity of U = 0.12 (m/s). From the temperature field, another vector field was generated by FIV [26], which successively cross-correlated the scalar fields to derive the underlying velocity field. The data sets used in this research are available for public download via Dryad [31] and are licensed under a CC0 1.0 Universal (CC0 1.0) Public Domain Dedication license. The software associated with the data set contains Matlab scripts that will reproduce the plots and tables within this paper and explore the data set. The software included in the data set can be used with Matlab R2022b, hosted by Zenodo and licensed under the standard MIT license [32].

## Abbreviations

The following abbreviations are used in this manuscript:

TSL	Thermal skin layer
VSL	Viscous skin layer
EMSL	Electromagnetic skin layer
DNS	Direct numerical simulation
FIV	Feature Image Velocimetry
PIV	Particle Image Velocimetry
TKE	Turbulent kinetic energy
TKED	Turbulent kinetic energy dissipation
LSM	Large-scale motion
FOV	Field of View
